# Multi-modal radiomics model based on four imaging modalities for predicting pathological complete response to neoadjuvant treatment in breast cancer

**DOI:** 10.1186/s12885-025-14407-2

**Published:** 2025-06-02

**Authors:** Yuwen Liang, Haonan Xu, Jie Lin, Wenqiang Tang, Xinlan Liu, Kunyuan Gan, Qiaodi Wan, Xiaobo Du

**Affiliations:** 1 Department of Oncology, National Health Commission Key Laboratory of Nuclear Technology Medical Transformation, Mianyang Central Hospital, 12 Changjiaxiang, Mianyang, Sichuan 621000 People’s Republic of China; 2 Department of Oncology, Affiliated Hospital of North Sichuan Medical College, Nanchong, Sichuan 637000 People’s Republic of China; 3 Sichuan Clinical Research Center for Radiation and Therapy, Mianyang Central Hospital, Mianyang, Sichuan 621000 People’s Republic of China; 4 Ultrasound department, Mianyang Central Hospital, School of Medicine, University of Electronic Science and Technology of China, Mianyang, Sichuan 621000 People’s Republic of China

**Keywords:** Multi-modal, Radiomics, Breast Cancer, Neoadjuvant treatment, Pathological complete response

## Abstract

**Objective:**

The radiomics model based on single imaging modality has been demonstrated as a promising approach for predicting the response to neoadjuvant treatment (NAT) in breast cancer. However, whether integrating multiple imaging modalities improve the performance of the radiomics model is undetermined. This study aims to develop a multi-modal radiomics model based on four imaging modalities, including ultrasound (US), mammography (MM), computed tomography (CT), and magnetic resonance imaging (MRI), for predicting pathological complete response (pCR) in breast cancer after NAT.

**Methods:**

Patients who underwent surgery after NAT from January 2019 to July 2023 were retrospectively studied. Univariate and multivariate analyses were performed to identify independent clinical risk factors for pCR. The radiomic features were extracted from the volume of interest on the four imaging modalities. The least absolute shrinkage and selection operator was used for developing radiomic signatures. The multi-modal radiomics model was developed by combining four radiomic signatures. The combined model was developed by combining clinical risk factors and four radiomic signatures. A nomogram was developed to visualize the combined model. Model performance was internally validated by using the five-fold cross-validation.

**Results:**

In total, 89 patients were included, with the pCR rate of 31.5% (28/89). Multivariate analyses identified PR status (OR = 4.450, 95% confidence interval [CI], 1.228–18.063, *P* = 0.028), HER2 status (OR = 9.95, 95% CI, 1.525–201.894, *P* = 0.044) and clinical T stage (OR = 0.253, 95% CI, 0.076–0.753, *P* = 0.016) were independent clinical risk factors for pCR. The AUCs and brier scores of the radiomic signatures of US, MM, CT, and MRI were 0.702 (95% CI: 0.583–0.821), 0.762 (95% CI: 0.660–0.865), 0.814 (95% CI: 0.725–0.903), 0.787 (95% CI: 0.685–0.889) and 0.198, 0.177, 0.165, 0.170 respectively. The performance of the multi-modal radiomics model was superior to all radiomic signatures with an AUC of 0.904 (95% CI: 0.838–0.970) and with the brier score of 0.111. After adding independent clinical risk factors, the performance of the combined model further improved, achieving an AUC of 0.943 (95% CI: 0.893–0.992) and a brier score of 0.082. The nomogram showed potential clinical value.

**Conclusion:**

The multi-modal radiomics model based on US, MM, CT, and MRI could accurately predict pCR in breast cancer after NAT, which was superior to all radiomic signatures. Incorporating clinical risk factors may further improve the performance of the muti-modal radiomics model, which could provide valuable information for guiding treatment decisions.

## Introduction

Breast cancer (BC) is the leading cause of cancer incidence worldwide, accounting for 2.3 million new cases or 11.7% of all cancer diagnoses. This presents a significant global health challenge [1].

Neoadjuvant treatment (NAT) offers several clinical advantages, including tumor downstaging, reduced metastatic potential, and enabling breast-conserving surgery [2]. Consequently, NAT is now the standard therapeutic approach for many BC subtypes, especially locally advanced breast cancer [3]. Moreover, patients who achieve a pathological complete response (pCR) following NAT typically have better long-term outcomes, including improved disease-free and overall survival [4].

Currently, the gold standard for evaluating NAT response is pathological examination of surgical specimens. According to the Miller-Payne grading system, pCR is defined as the absence of residual invasive tumor cells in both the breast and axillary lymph nodes (ypT0/is + ypN0) [5]. However, this method is inherently retrospective and can only guide treatment adjustments after surgery. Predicting therapeutic responses early, before initiating NAT, could transform clinical decision-making by enabling personalized treatment plans and sparing non-responders from unnecessary toxicity. As a hybrid modality that integrates both metabolic and anatomical features, PET/CT has demonstrated significant potential in predicting NAT response in breast cancer and is mentioned in the most updated guidelines [6, 7].

Radiomics, an emerging field at the intersection of imaging and computational science, offers a promising approach for characterizing tumor heterogeneity and predicting treatment outcomes non-invasively by extracting numerous quantitative features from medical images [8]. Radiomics can serve as a bridge connecting tumor heterogeneity and imaging manifestations, making it a non-invasive biomarker for BC diagnosis and prognosis [9]. The progress in computer science has greatly increased the potential of radiomics [10]. Recent advancements in artificial intelligence techniques, such as machine learning and deep learning, have significantly improved the applicability and generalizability of radiomics in various medical fields [11]. In clinical practice, imaging modalities such as ultrasound (US), computed tomography (CT), mammography (MM) and PET/CT are commonly employed to assess the extent of breast tumors and axillary lymph node involvement. Recent studies have shown that radiomics models based on single imaging modalities can predict responses to NAT [12, 13]. However, single-modality radiomics models may fail to capture the complex heterogeneity of tumors, including spatial, biological, and genetic variability [14]. Multi-modal radiomics models, which combine features from various imaging modalities, may provide more valuable information to overcome this limitation. This study aims to develop and validate a multi-modal radiomics model integrating four imaging modalities—US, MM, CT, and MRI—to predict pCR in BC after NAT.

## Materials and methods

### Study design

**Fig. 1:**
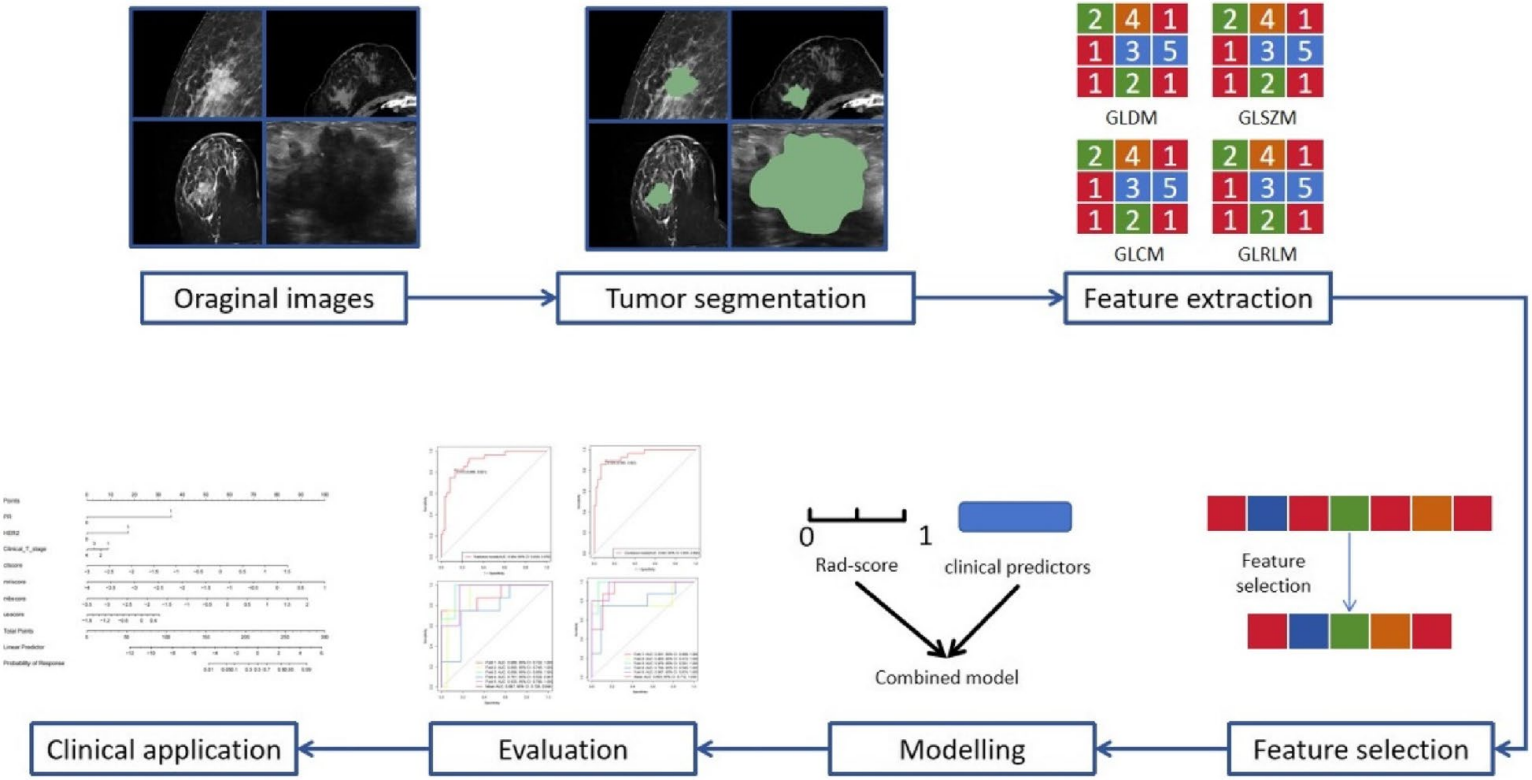
Workflow of mut-modal radiomics model for pCR prediction in patients with locally advanced breast cancer. pCR, pathological complete response

This single-center retrospective study was conducted in accordance with the Declaration of Helsinki and approved by the Ethics Committee of Mianyang Central Hospital. Informed consent was waived due to the retrospective design of the study. Surgically resected specimens served as the reference standard for assessing treatment response. The study aims to develop and validate a multi-modal radiomics model to predict pCR in BC following NAT. The combined model integrates pretreatment imaging features from US, MM, CT, and MRI, along with clinical risk factors (Fig. 1).

### Patients

The inclusion criteria for this study were as follows:

1. Patients with biopsy-confirmed BC without distant metastasis.
2. Received NAT without prior anti-tumor treatment.
3. Completed pretreatment imaging, including breast US, MRI, MM, and CT, at Manyang Central Hospital.
4. Underwent surgery after completing NAT.

The exclusion criteria included:

1. Patients with incomplete or low-quality imaging data (e.g., significant motion artifacts).
2. Did not complete NAT or received non-standard treatments.
3. With a history of prior malignancies or other concurrent tumor diseases.
4. Aged under 18 years.
5. Patients who underwent biopsy or surgery at external institutions without available pathological confirmation of response.

### Neoadjuvant chemotherapy and pathological assessment of response

All patients received 4, 6, or 8 cycles of NAT before breast surgery. The treatment regimen and timeline adhered to the National Comprehensive Cancer Network (NCCN) guidelines. NAT regimens included taxane-based, anthracycline-based, or combination anthracycline and taxane-based therapies [15]. Trastuzumab was included in the treatment for HER2-positive patients. The trastuzumab dosing regimen consisted of an 8 mg/kg loading dose, followed by a 6 mg/kg maintenance dose.

A pathological assessment was performed on surgically resected specimens following NAT. Tissue samples were fixed in 10% neutral-buffered formalin and processed overnight. Sections were cut at a thickness of 5 μm thickness and stained using automated staining systems. Experienced breast pathologists, each with at least 5 years of expertise, conducted the assessments. Pathologists were blinded to the imaging data to minimize bias. In cases of disagreement, results were reviewed and resolved through consensus discussions among pathologists. pCR was defined as the absence of invasive carcinoma in both the breast and axillary lymph nodes. Residual ductal carcinoma in situ was permitted (ypT0/isN0) [16, 17].

### Image data acquisition and segmentation

In this study, breast US, MM, MRI, and chest CT images were obtained within two weeks before NAT in BC patients. All images were retrospectively retrieved from the Picture Archiving and Communication System (PACS) at our institution and stored in Digital Imaging and Communications in Medicine (DICOM) format. For US, CT, and MRI, the image showing the maximum tumor diameter was selected. MM images were selected from the mediolateral oblique (MLO) view, which provided the clearest depiction of the tumor. Axial chest CT scans were acquired using standard protocols. The axial fat-suppressed T2-weighted imaging (T2 WI) sequence was obtained before contrast medium administration. Regions of interest (ROI) for each image were manually delineated by experienced radiologists using 3D Slicer software (version 4.10 from https://www.slicer.org/) [18]. The ROIs included only the tumor. The radiologist was blinded to the pathological and clinical data.

### Features extraction

The PyRadiomics (version 3.0.1, http://github.com/Radiomics/pyradiomics#readme) package of Python software (version 3.9.7) was used to extract features from the volumes of interest (VOIs) in the US, MM, CT, and MRI images. These features included several categories, such as Shape2D, First Order Statistics, Shape, Gray Level Co-occurrence Matrix (GLCM), Gray Level Size Zone Matrix (GLSZM), Gray Level Run Length Matrix (GLRLM), Gray Level Dependence Matrix (GLDM), and Neighboring Gray Tone Difference Matrix (NGTDM). Detailed definitions of these radiomics features are available in prior documentation [19] and can be accessed at https://pyradiomics.readthedocs.io/en/latest/ internal-validation/features.html.

### Features selection and model building

The least absolute shrinkage and selection operator (LASSO) method, which is suitable for high-dimensional data regression [20], was used to select the most robust predictive features. A radiomics signature was then constructed, and the radiomics score (Rad-score) for each patient was calculated as a linear combination of selected features weighted by their respective LASSO coefficients. The multi-modal radiomics model was developed by combining four radiomics signatures.

Univariate and multivariate analysis was used to identify independent clinical predictors associated with pCR. Multivariate analysis included only factors with a *P* value < 0.05 from univariate analysis. The combined model was developed using logistic regression with independent clinical predictors and four Rad-scores, and a nomogram was created to visualize the model.

### Assessment of radiomics model predictive performance

The area under the curve (AUC) of the receiver operating characteristic (ROC) curve was used to assess the discrimination of the multi-modal radiomics model and combined model. Cross-validation is a widely used method to construct validation sets, effectively mitigating overfitting and underfitting while enhancing model generalizability [21, 22], particularly for small-sample datasets [23]. The performance of the radiomics model was validated using five-fold cross-validation.

### Statistical analyse

The statistical analyses were performed using R software version 3.6.1 (http://www.Rproject.org). The statistical analyses were carried out using the R software Quantitative data were described as means ± standard deviations, and qualitative data were described as frequencies (percentages). The independent predictive factors in the variables are determined based on multiple regression analysis. Significant difference was based on P < 0.05. The “glmnet” package was used in LASSO regression analysis, and the “rms” package was used in univariate and multivariate analysis. The “pROC” package was used to process the collected data and establish the ROC diagram. The nomogram was plotted using the “rms” package. Statistical significance was two-sided, with significance level at *P* < 0.05.

## Results

### Clinical characteristics

**Fig. 2:**
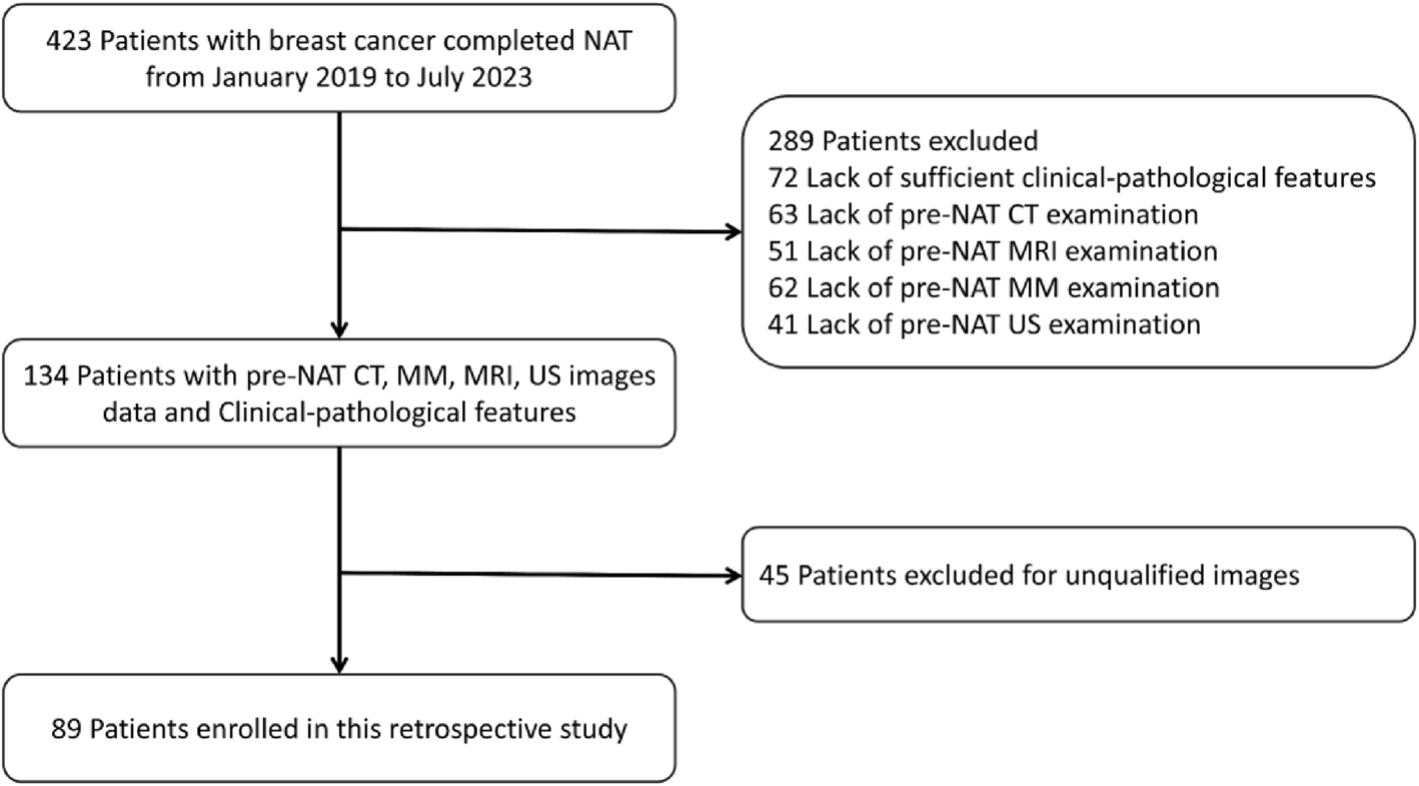
Flow chart of the study. NAT, neoadjuvant treatment; US, ultrasonography; CT, computed tomography, MRl, magnetic resonance imaging; MM,mammography, US, ultrasound radiomic signatures

This study reviewed the clinical and pathological characteristics of 423 BC patients at Sichuan Mianyang Central Hospital from January 2019 to July 2023. A total of 334 patients were excluded according to the exclusion criteria. Of these, 72 patients lacked complete clinical or pathological information, 217 patients did not undergo at least one required pretreatment imaging examination (US, MM, CT, or MRI), and 45 patients were excluded due to poor imaging quality. Ultimately, 89 patients were included in the subsequent study (Fig. 2).

Among the 89 included patients, 28 achieved the pCR, with a pCR rate of 31.46%. All patients were female. The mean ages were 50.10 ± 9.23 for all patients. Detailed patient characteristics are summarized in Table 1.

**Table 1:** Characteristics of patients with pCR and non- pCR

	non-pCR *n* = 61	pCR *n* = 28	*p*
Age (year, mean ± SD)	50.77 ± 9.21	48.64 ± 9.27	0.889
Menopausal status (%)			0.700
0	30(49.18)	15(53.57)	
1	31(50.82)	13(46.43)	
ER Status (%)			0.022
Negative	31(50.82)	7(25.00)	
Positive	30(49.18)	21(75.00)	
PR Status (%)			0.003
Negative	46(75.41)	12(42.86)	
Positive	15(24.59)	16(57.14)	
HER2 Status(%)			0.023
Negative	14(22.95)	1(3.57)	
Positive	47(77.05)	27(96.43)	
Ki-67 Status (%)			0.214
Negative	22(36.07)	14(50.00)	
Positive	39(63.93)	14(50.00)	
cT (%)			0.003
1	0(0)	2(7.14)	
2	25(40.98)	19(67.86)	
3	36(59.02)	7(25.00)	
cN (%)			0.482
0	19(31.15)	13(46.43)	
1	32(52.46)	11(39.29)	
2	7(11.48)	2(7.14)	
3	3(4.92)	2(7.14)	
Stage (%)			0.005
II	31(50.82)	23(82.14)	
III	30 (49.18)	5(17.86)	

The data is presented as numbers and percentages [*n* (%)]. The *p* values were calculated by the chi-square test. Ki-67 Status: Ki-67 status negative: Ki-67 index ≤ 20%, Ki-67 status positive: Ki-67 index > 20%

The clinical and pathological features of the 89 patients were analyzed to identify factors associated with pCR after NAT. Univariate analysis identified that the factors related to pCR included estrogen receptor (ER) status, progesterone receptor (PR) status, human epidermal growth factor receptor 2 (HER2) status, clinical T stage and stage. No statistically significant differences were found for age, menopausal status, Ki-67 index and clinical N stage (*P* > 0.05). The multivariate analysis revealed that PR status (OR = 4.450, 95% confidence interval [CI], 1.228–18.063, *P* = 0.028), HER2 status (OR = 9.95, 95% CI, 1.525–201.894, *P* = 0.044) and clinical T stage (OR = 0.253, 95% CI, 0.076–0.753, *P* = 0.016) were independent clinical predictors of pCR (Table 2).

**Table 2:** Univariate and multivariable analysis

Variable		OR		95% CI	*P*
Age		0.97		0.93–1.02	0.312
Menopausal status		0.84		0.34–2.06	0.701
ER Status		3.10		1.19–8.86	0.025
PR Status		4.09		1.61–10.82	0.004
HER2 Status		8.04		1.49–149.73	0.050
Ki-67 Status		0.56		0.23–1.40	0.216
cT		0.22		0.08–0.54	0.002
cN		0.799		0.43–1.37	0.417
Stage		0.2		0.07–0.63	0.007
Univariate analysis					
Variable	OR		95% CI		*P*
ER Status	1.14		0.29–4.46		0.847
PR Status	4.45		1.23–18.04		0.028
HER2 Status	9.95		1.52—201.89		0.044
cT	0.25		0.07–0.75		0.016
Stage	0.39		0.10–1.45		0.164
Multivariable analysis					

### Radiomics signature construction

**Fig. 3:**
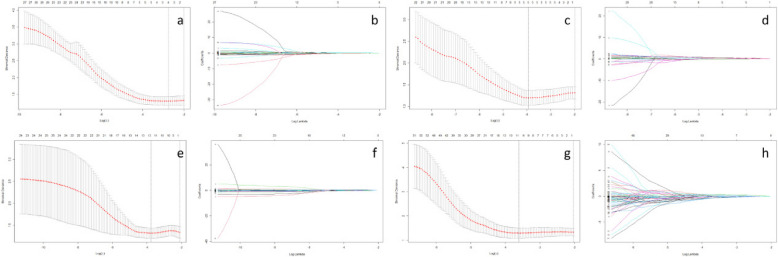
Radiomic feature selection using the least absolute shrinkage and selection operator (LASSO) logistic regression model. (**a**)(**c**)(**e**)(**g**) Identification of the optimal penalization coefficient lambda (λ) in the LASSO model used tenfold cross-validation and the minimum criterion. As a result, a λ value of 0.026817 was selected. (**b**) LASSO coefficient profiles of 5 features selected among 27 features based on US images. (**d**) LASSO coefficient profiles of 4 features selected among 27 features based on MM images. (**f**) LASSO coefficient profiles of 11 features selected among 30 features based on CT images. (**h**) LASSO coefficient profiles of 10 features selected among 278 features based on MRI images

In total, radiomics features were extracted from each patient based on US, MM, CT and MRI images were 474, 474, 851 and 851 respectively. For further selection, the t-test was conducted to filter the features with statistically significant differences (P < 0.05) between pCR cohort and non-pCR cohort and drew 27, 27, 30, 278 filtered features respectively. Among them, 4, 5, 11 and 10 features with the highest predictive value were selected as final predictors using the LASSO regression algorithm (Fig. 3). The Rad-score for each imaging modality was calculated by a weighted linear combination of these twelve features and their corresponding coefficients. The multi-modal radiomics model was developed by combining the four radiomic signatures.

The Rad-scores of each imaging modality were as follows:

US-score = −0.798–0.076 × original_shape2D_MaximumDiameter-0.152 × original_glrlm_RunLengthNonUniformity + 0.198 × wavelet.HL_ngtdm_Coarseness −0.005 × wavelet.LL_glrlm_RunLengthNonUniformity.

MM-score = −0.904 + 0.609 × original_shape2D_Sphericity + 0.478 × original_firstorder_10Percentile + 0.164 × original_glcm_Imc2-0.248 × wavelet.HH_glcm_Imc2 + 0.102 × wavelet.LL_glcm_Imc2.

CT-score = −0.928–0.149 × original_glrlm_LongRunHighGrayLevelEmphasis-0.197 × wavelet.LLH_glszm_SizeZoneNonUniformity-0.278 × wavelet.LHH_glszm_SizeZoneNonUniformity + 0.260 × wavelet.HLH_gldm_HighGrayLevelEmphasis-0.001 × wavelet.HLH_gldm_LowGrayLevelEmphasis-0.199 × wavelet.HLH_glszm_SmallAreaEmphasis-0.254 × wavelet.HHL_glszm_SmallAreaLowGrayLevelEmphasis-0.151 × wavelet.HHL_glszm_ZoneEntropy-0.248 × wavelet.HHH_firstorder_Median + 0.246 × wavelet.HHH_glcm_ClusterProminence-0.169 × wavelet.HHH_ngtdm_Complexity.

MRI-score = −0.940–0.064 × wavelet.LLH_gldm_SmallDependenceLowGrayLevelEmphasis-0.317 × wavelet.LLH_glszm_SmallAreaLowGrayLevelEmphasis + 0.104 × wavelet.LHL_gldm_SmallDependenceHighGrayLevelEmphasis-0.308 × wavelet.LHL_gldm_SmallDependenceLowGrayLevelEmphasis-0.279 × wavelet.LHL_ngtdm_Busyness + 0.353 × wavelet.HLH_firstorder_RootMeanSquared + 0.011 × wavelet.HLH_glcm_InverseVariance + 0.024 × wavelet.HLH_glrlm_RunLengthNonUniformityNormalized + 0.252 × wavelet.HHH_glcm_InverseVariance-0.087 × wavelet.HHH_glrlm_LongRunLowGrayLevelEmphasis.

### Radiomics signature predictive performance`

**Fig. 4:**
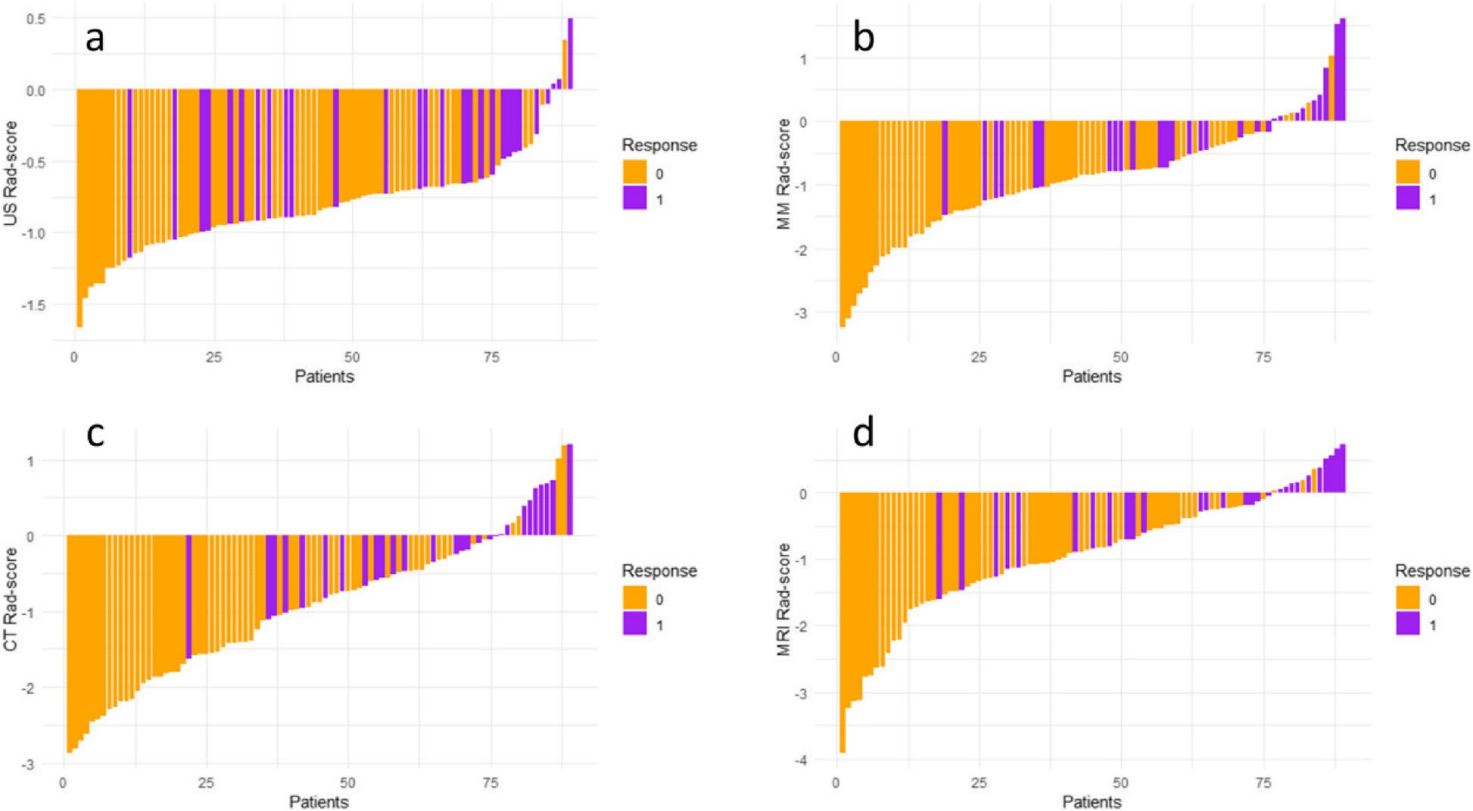
Rad-scores in pCR group and non-pCR group. (**a**) US-score. (**b**) MM-score. (**c**) CT-score. (**d**) MRI-score. The orange bars indicate the patients in the non-responders group, while the purple bars represent patients in the responders group

**Fig. 5:**
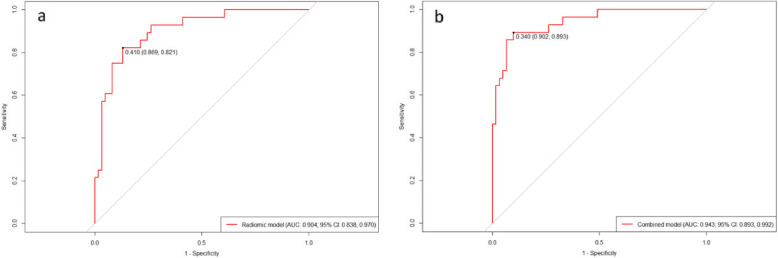
Receiver operating characteristic curves of the mut-modal radiomics model (**a**) and combined model (**b**). AUC, area under the receiver operator characteristic curve; CI. confidence interval

The AUCs of the radiomic signatures for US, MM, CT, and MRI were 0.702 (95% CI: 0.583–0.821), 0.762 (95% CI: 0.660–0.865), 0.814 (95% CI: 0.725–0.903), 0.787 (95% CI: 0.685–0.889), respectively. The brier scores for the radiomic signatures of four imaging modalities above were 0.198, 0.177, 0.165, 0.170, respectively. The waterfall plot showed the distribution of Rad-scores and response for all patients. Patients with higher Rad-scores appeared are more likely to achieve pCR than those with lower Rad-scores (Fig. 4). Among the predictive models developed by single imaging modalities, the radiomics signature derived from CT images demonstrated the best predictive performance. Furthermore, the multi-modal radiomics model outperformed all individual radiomic signatures with an AUC of 0.904 (95% CI: 0.838–0.970) and with a Brier score of 0.111 (Fig. 5a).

To further analyze the importance of four imaging modalities in predicting pCR after NAT in BC, this study constructed multi-modal radiomic models by integrating three different single-modality radiomic features, with their AUC values presented in Table 3. The results indicated that, compared to the multimodal radiomics model, the three-modality prediction model excluding CT-based radiomic features exhibited the most significant decrease in AUC, followed by the model excluding MRI-based radiomic features. Additionally, the results demonstrated that the AUC of the multimodal radiomics model was superior to all three-modality radiomics models, though the differences in sensitivity and specificity were not significant.

**Table 3:** Multi-modal radiomic models by integrating three different single-modality radiomic features

Modeling Modalities	Excluded Modality	AUC (95% *CI)*	Sensitivity	Specificity	AUC Increment
US MM CT MRI	-	0.904 (0.838–0.970)	0.821	0.869	-
MM CT MRI	US	0.899 (0.831–0.967)	0.750	0.918	0.005
US CT MRI	MM	0.882 (0.806–0.958)	0.750	0.885	0.022
US MM CT	MRI	0.872 (0.780–0.945)	0.964	0.705	0.032
US MM MRI	CT	0.865 (0.785–0.944)	0.786	0.820	0.039

### Combined model construction and performance assessment

**Fig. 6:**
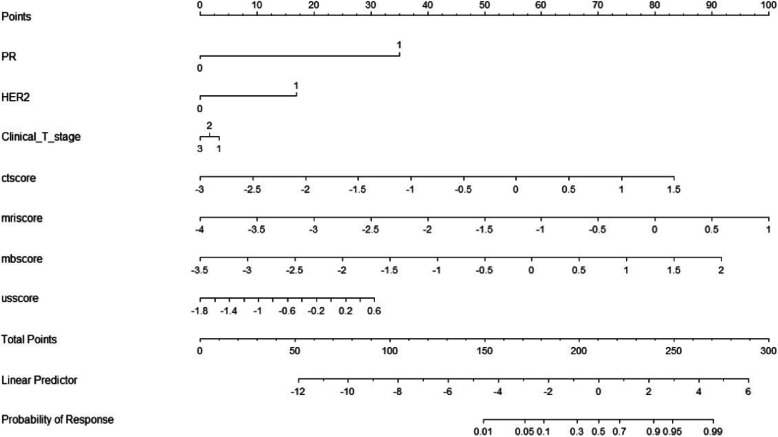
The nomogram for predicting the probability of pCR. PR, progesterone receptor, HER-2, human epidermal growth factor receptor2; CT score, computed tomography radiomic signatures, MRl score, magnetic resonance imaging radiomic signatures; MMscore,mammography radiomic signatures, US score, ultrasound radiomic signatures, pCR, pathological complete response

A combined model was developed using the three independent clinical predictors and four radiomics signatures to estimate the risk of pCR. The combined model showed good discrimination with an AUC of 0.943 (95% CI: 0.893–0.992) and a Brier score of 0.082 (Fig. 5b). A nomogram was developed to visualize the model. The total score was calculated based on seven variables: PR status, HER2 status, clinical T stage, CT-score, MRI-score, MM-score, and US-score. Each score was summed and projected onto the total point scale to estimate the predicted probability. This nomogram can be easily applied in clinical practice for visual risk estimation (Fig. 6). For example, consider a patient with PR-positive status (35 points), HER2-positive status (17 points), clinical T1 stage (3 points), CT-score of −1 (36 points), MRI-score of −1.5 (50 points), MM-score of 0 (57 points), and US-score of 0 (22 points). The above variables yielded a total score of 220 points for this patient, with a corresponding possibility of pCR of approximately 70%.

### Validation of the multi-modal radiomics model and the combined model

**Fig. 7:**
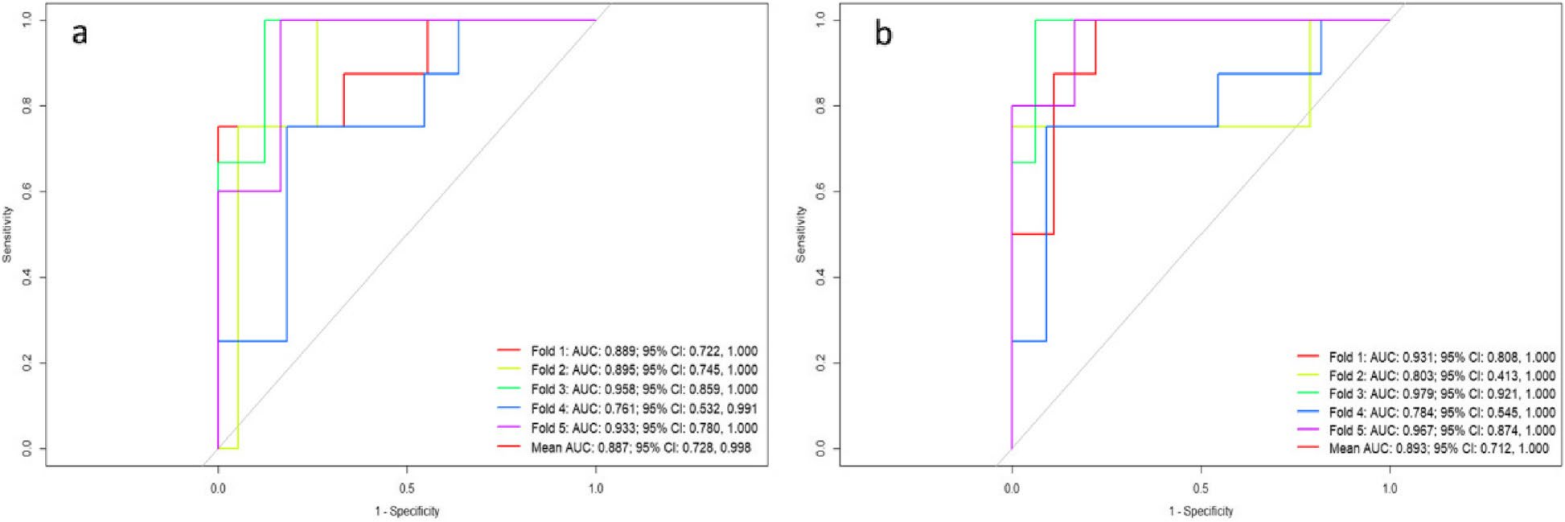
Validation of the multi-modal radiomics model and the combined model. (**a**) Multi-modal radiomics model. (**b**) Combined model. AUC, area under the receiver operator characteristic curve; CI, confidence interval

Internal five-fold cross-validation results showed that both the multi-modal radiomics model and the combined model exhibited good predictive performance, with mean AUCs of 0.887 (95% CI: 0.728–0.998) and 0.893 (95% CI: 0.712–1.000), respectively (Fig. 7). Incorporating clinical predictors similarly improved the predictive performance of the multi-modal radiomics model.

## Discussion

This study developed a multi-modal radiomics model utilizing four imaging modalities—US, MM, CT, and MRI—to predict the pCR in BC patients after NAT. The results indicate that the multi-modal radiomics approach demonstrates strong predictive performance, with an AUC of 0.887 (95% CI: 0.728–0.998) in the internal cross-validation set. The model exhibited a sensitivity of 0.82, specificity of 0.87, and accuracy of 0.74. This preliminary validation suggests that the radiomics model integrating multiple imaging modalities outperforms single-modality radiomics models in predicting pCR after NAT.

In the clinical management of BC, NAT has become an essential component of standard treatment strategies. However, due to the heterogeneity and complexity of tumors, different patients—despite being at the same stage and sharing similar clinicopathological characteristics—may exhibit significant variations in their responses to NAT. This variability affects the emphasis of treatment strategies [24, 25]. Currently, the efficacy of NAT in BC patients is assessed through postoperative pathological examination of surgical specimens, with histopathological analysis of resected specimens considered the gold standard [26]. However, this approach has a time lag, complicating clinicians'ability to preoperatively identify patients who are likely to have a poor or no response to NAT, thus hindering timely treatment intensification. The Response Evaluation Criteria in Solid Tumors (RECIST) are commonly used to assess the response to NAT by measuring changes in the maximum tumor diameter on US, MM and MRI [27, 28]. Typically, after NAT, tumor cells become hypoxic and fragment, leaving behind fibrosis and collagenous tissue. As a result, even when the tumor volume is significantly reduced, the tumor diameter may remain unchanged. This makes it challenging for conventional imaging techniques to accurately reflect changes in the tumor microenvironment [29]. In contrast to traditional methods, radiomics, an emerging technology, extracts high-throughput quantitative features from medical images, facilitating enhanced diagnosis and prognostic assessment [30].

Radiomics features extracted from US, MM,MRI and PET/CT have been extensively studied as potential biomarkers for predicting NAT efficacy. For instance, Jiang et al. [31] developed a predictive model based on pre-NAT breast US images, achieving an AUC of 0.82 in the validation set—comparable to the performance of the model presented in this study. In comparison, the single-modality radiomics model based on US images in this study achieved an AUC of 0.72. It's worth noting that Jiang et al. [31] employed deep learning techniques for their predictive model, while this study utilized the LASSO regression method. These differing modeling approaches may account for the performance variation between the two models. MRI provides multiparametric imaging sequences that can capture the biological and pathological characteristics of lesions from multiple perspectives, including morphology and metabolism. Therefore, radiomics features extracted from MRI images are frequently utilized in predictive modeling. Parikh et al. [32] extracted radiomics features from T2 WI and T1 WI MRI sequences to construct predictive models. Their findings indicated that the model based on T2 WI images significantly outperformed the T1 WI-based model in predicting pCR among BC patients (maximum sensitivity: 87.5% vs. 50%), while specificity remained consistent. In a multicenter study by Liu et al. [12], a predictive model based on multiparametric MRI images was developed, with the T2 WI-based model achieving an AUC of 0.69 for pCR prediction. Our study's T2 WI MRI-based predictive model achieved an AUC of 0.79, aligning with Liu et al.'s findings and further supporting the reliability of radiomics features derived from T2 WI MRI images in predicting pCR. MM is also one of the commonly used imaging modalities for BC screening and diagnosis, with radiomics models based on MM images showing promising predictive performance [33]. Skarping et al. [34] developed a predictive model based on MM images, achieving an AUC of 0.71, with a sensitivity of 46% at a specificity of 90%. Similarly, the MM-based predictive model in our study achieved an AUC of 0.76, demonstrating good predictive capability. Radiomic features extracted from PET/CT images have been explored as potential biomarkers for evaluating therapeutic responses in various oncological diseases, particularly in lung cancer and breast cancer [6, 35]. Despite the increasing recognition and integration of this technology in clinical practice, the application of PET/CT radiomics in our study was limited due to the small number of patients at our institution who underwent PET/CT imaging.

Different imaging modalities have their respective advantages, and in clinical practice, multiple imaging examinations are often employed to comprehensively assess BC patients from various perspectives. Extracting radiomics features from multiparametric MRI images is a common approach for building multi-modal radiomics models. For example, Bian et al. [36] developed a radiomics model to predict pCR after NAT by extracting radiomics features from dynamic contrast-enhanced (DCE), T2 WI, and DWI MRI sequences in BC patients. This model achieved an AUC of 0.91, with an accuracy of 88.9%, sensitivity of 88.2%, and specificity of 90.9%. Similarly, studies conducted by Liu et al. [12], Chen et al. [37], and Xiong et al. [25] on radiomics models based on multiple MRI sequences have also shown that multi-modal radiomics models constructed from multiparametric MRI images can effectively predict BC patients’ responses to NAT, with reported AUCs ranging from 0.79 to 0.94.

Another widely used approach for developing multi-modal radiomics models is the integration of imaging features from various imaging modalities. Montemezzi et al. [38] developed a multi-modal radiomics model by combining radiomics features extracted from pre-NAT ^18^F-FDG PET-CT and DCE-MRI images. Their study showed that incorporating features from both imaging modalities improved the model’s predictive performance compared to single-modality models, achieving an AUC of 0.96. Urso et al. [39] utilized machine learning techniques to create radiomics models using baseline 18 F-FDG PET-CT and CT images. Their findings demonstrated that the multi-modal model, which incorporated both FDG PET and CT features, significantly outperformed the CT-only model, achieving a maximum AUC of 0.79 compared to 0.59 for the CT-only model. These results underscore the potential of multi-modal radiomics in improving predictive accuracy.

In another study, Cai et al. [40] constructed a multi-modal radiomics model based on pre-treatment US and MM images to predict pCR in BC patients following NAT. Their model outperformed both a model that integrated only clinical features and a single-modality mammography-based model, with AUCs of 0.81 (95% CI: 0.75–0.87) compared to 0.79 (95% CI: 0.72–0.85). This multi-modal radiomics model, which utilized features from four imaging modalities, exhibited superior predictive performance compared to all single-modality models. This finding suggests that incorporating radiomics features from additional imaging modalities may further enhance the predictive performance of the model.

Studies have indicated that clinical and pathological characteristics of BC patients, such as tumor size, tumor grade, pathological type, and hormone receptor status, can serve as predictive factors for response to NAT [41–44]. Univariate and multivariate analyses in one study identified PR status (P = 0.028), HER2 status (P = 0.044), and clinical T stage (P = 0.016) as independent clinicopathological predictors of pCR in BC patients following NAT. Hormone receptor status is a well-established pathological factor influencing treatment response and prognosis in breast cancer. Several studies, including those by Liu et al. [12], Wang et al. [45], and Mao et al. [46], have found significant associations between hormone receptor status, tumor TNM stage, and pCR after NAT. Nonetheless, relying solely on pathological characteristics does not yield satisfactory predictive performance [47]. This limitation may stem from the inherent difficulty of manually distinguishing tumor tissue from fibrotic tissue on imaging scans. When a tumor responds to NAT, both the tumor and its microenvironment undergo changes that are challenging to detect with conventional assessments.

Nevertheless, combining clinicopathological features with radiomics features has been shown to further enhance predictive performance. A meta-analysis by Liang et al. [48], which included 17 studies involving 3,392 patients, demonstrated that radiomics models integrating clinical or histopathological features outperformed models based solely on MRI image features (AUC: 0.90 vs. 0.82). The findings of our study are consistent with these results, as the combined model that included clinicopathological features achieved a higher AUC than the multi-modal radiomics model based solely on radiomics features (0.893 vs. 0.887).

This study employed internal five-fold cross-validation to assess the predictive performance of the model. In the context of a limited sample size, resampling strategies play a crucial role [49]. The primary goal of cross-validation is to reduce overfitting and provide a more accurate estimation of the predictive performance of radiomics-based models on new datasets [50]. This approach maximizes data utilization while minimizing the variability of performance estimates caused by random data splitting. Similar studies by Yu [51] and Meng [52] have also employed internal cross-validation to validate the predictive capabilities of radiomics models, yielding results consistent with those of this study. These findings suggest that internal cross-validation is a reliable method for evaluating model performance when the sample size is relatively small.

The limitations of this study are as follows: First, this study is a single-center, small-sample, retrospective study, which may introduce unavoidable selection bias. Future research should expand the cohort, incorporate multi-center data for model training, and, whenever possible, validate model performance using external datasets. However, the primary objective of this study was to determine whether a multimodal radiomics model outperforms single-modality radiomics models. After obtaining preliminary validation, further investigations with larger cohorts are warranted. Second, the image segmentation process in this study was performed manually, which is both time-consuming and labor-intensive. Moreover, manual segmentation may be influenced by the experience of the radiologists. With advancements in deep learning and artificial intelligence, semi-automatic and fully automated segmentation techniques have been continuously improving. Future studies should focus on refining ROI delineation methods to enhance reproducibility and efficiency [53, 54]. Third, regarding the direct comparison of predictive performance between single- and multi-modality models, as this study employed internal five-fold cross-validation for model validation, the reported AUC values represent the average AUC across the folds. Therefore, it was not feasible to conduct a direct statistical comparison using methods such as the DeLong test. In future large-sample studies, this issue can be addressed by utilizing an independent internal or external validation cohort. Fourth, various machine learning algorithms, such as random forest and support vector machines, are increasingly being applied in radiomics research. This study used the LASSO regression as a basic modeling approach to preliminarily validate the improved predictive performance of the multimodal radiomics model over single-modality models. Future studies should explore multiple machine learning algorithms for model development to identify optimal modeling approaches and further enhance model performance.

## Conclusions

This study preliminarily validated that multimodal radiomics outperforms single-modal radiomics models in accurately predicting pCR after NAT in BC patients. Future studies should include larger, multi-center cohorts to validate the generalizability of this model. Prospective validation is also necessary to confirm our findings and ensure the clinical applicability of the model in predicting treatment outcomes.

## Data Availability

Data is provided within the manuscript or supplementary information files.
